# Therapeutic implications of exosomes in the treatment of radiation injury

**DOI:** 10.1093/burnst/tkab043

**Published:** 2022-01-21

**Authors:** Shijie Dai, Yuzhong Wen, Peng Luo, Le Ma, Yunsheng Liu, Junhua Ai, Chunmeng Shi

**Keywords:** Exosome, Radiation injury, Treatment

## Abstract

Radiotherapy is one of the main cancer treatments, but it may damage normal tissue and cause various side effects. At present, radioprotective agents used in clinics have side effects such as nausea, vomiting, diarrhea and hypotension, which limit their clinical application. It has been found that exosomes play an indispensable role in radiation injury. Exosomes are lipid bilayer vesicles that carry various bioactive substances, such as proteins, lipids and microRNA (miRNA), that play a key role in cell-to-cell communication and affect tissue injury and repair. In addition, studies have shown that radiation can increase the uptake of exosomes in cells and affect the composition and secretion of exosomes. Here, we review the existing studies and discuss the effects of radiation on exosomes and the role of exosomes in radiation injury, aiming to provide new insights for the treatment of radiation injury.

Highlights

- This article reviews the effects of radiation on the biological effects of exosomes and the role of exosomes in radiation-induced skin injury, radiation-induced lung injury and radiation-induced bone injury.
- Exosomes provide new insights for the treatment of radiation injury.

## Background

The source of radiation exposure is related to medical procedures, such as clinical diagnosis and radiotherapy, and industry activities [1]. Radiotherapy is one of the major cancer treatments. Although radiotherapy can improve the local control rate of tumours and patients outcomes, normal tissues often show radiation injury following radiotherapy [2]. Radiotherapy lead to various side effects, such as radiation-induced skin fibrosis, lung injury, bone loss, etc. In addition, radiation-induced bystander effects (RIBE) in unexposed tissues has shown phenomena similar to radiation exposure, such as apoptosis, gene instability, gene expression changes, chromosome breakage and mutations [3]. Serious injury can influence treatment and restrict the effective therapeutic dose for tumour control [4]. Therefore, urgent issues for cancer patients receiving radiotherapy include the ancillary antitumour effect and resistance to radiation injury [5]. The two main mechanisms that lead to radiation-induced tissue injury are direct DNA injury and reactive oxygen species (ROS) production [6]. Ionizing radiation (IR) absorbed by tissues affects molecules in cells. The absorbed energy gives rise to free radicals in the cells, most commonly ROS that are highly reactive. Excessive ROS leads to DNA injuries, such as simple base damage, DNA double-strand breaks (DSBs) or DNA crosslinks [7,8]. These injuries induce several cellular repair mechanisms, e.g. base excision repair and homologous recombination. However, if massive DNA injury is induced, these repair mechanisms fail, eventually leading to cell cycle arrest, senescence or cell death [9]. Thus, radiation-induced tissue injury has become a focus of prevention and treatment in biomedical studies. Amifostine (WR-2721) remains the only clinically approved broad-spectrum radioprotector that can clear out free radicals, protect DNA and accelerate repair [10]. However, the radioprotector’s toxic side effects, such as nausea, vomiting, diarrhoea and hypotension [10,11], limit its clinical use.

In recent years, a growing number of studies have found that exosomes play a significant role in radiation injury, which has aroused great interest among researchers. With an average diameter of ~100 nm [12], exosomes are common membrane-bound nanovesicles that carry various bioactive substances, such as lipids, proteins and nucleic acids, which are involved in intercellular communication and cellular signal transduction and changes in cell or tissue metabolism [13]. In addition, exosomes are also involved in the immune response [14] and the rescue of apoptosis [15]. Furthermore, recent studies have reported the important role of exosomes in disease treatment, including cancer [16], cardiovascular diseases [17,18], neurodegenerative diseases [19] and tissue injury [20]. A number of clinical trials have confirmed the safety of using exosomes in humans for immunotherapy [21,22]. As cell-free therapeutic drugs, exosomes, as endogenous vesicles, usually do not cause an immune response when entering humans [23], so they are considered to be safer than cell therapy. Moreover, exosomes have almost no cytotoxicity and have better storage stability and antiserum aggregation ability [24]. In addition, exosomes can be stored stably for a long period at −80°C. In regenerative medicine, exosomes may be more advantageous than stem cells because they avoid the challenges and limitations associated with stem cells [25]. It has been proved that the survival rate of transplanted stem cells in ischemic tissue is low [26]. Moreover, the risks of cell dedifferentiation, immune rejection and tumour formation further limits the direct application of stem cell transplantation for treatment [27]. Furthermore, because of their bilayer membrane and nanoscale size, exosomes can preserve their cargoes from removal or damage caused by complement binding or macrophages, extending their circulating half-life and increasing their biological activity [28]. Thus, exosomes as carriers have great application potential. Hazawa *et al*. [29] found that radiation enhanced cell uptake of exosomes by forming the tetraspanin complex CD29/CD81, suggesting the potential role of exosomes in radiation injury. Furthermore, exosomal microRNA (miR-210) can regulate the transcription pattern of hypoxia-inducible factor-1 to promote a more effective repair of genomic DSBs, thereby enhancing the radioresistance of cells [30]. In this review, we discuss the effects of radiation on the biological effects of exosomes and the role of exosomes in radiation-induced skin, lung and bone injury, which provides new insights into the treatment of radiation injury.

## Review

### Exosomes

The term ‘exosome’ was put forward as early as the 1980s when exosomes were originally thought of as simple molecular trash cans for packaging and transporting waste components in cells [31]. Exosomes exist in various extracellular fluids, including urine, saliva, blood, plasma, amniotic fluid, breast milk, cerebrospinal fluid etc. [32–34]. They have a typical cup-shaped morphology with negative staining, but they are round when viewed by transmission electron microscope and cryo-electron microscope [35,36]. Furthermore, their secretion occurs naturally, and cellular stress and activation signals can regulate the related processes [37]. Previous studies have found that exosomes derived from B lymphocytes can induce antigen presentation effects while motivating T lymphocyte activity [38]. Ever since then, researchers have been extremely interested in the study of exosomes. Studies have found that exosomes isolated from diverse body fluids show significant morphological multiplicity, indicating subsets of exosomes with diverse functions and contents [39,40]. In one study, researchers analyzed and quantified the morphology of exosomes secreted by the human mast cell line HMC-1 through electron microscopy techniques and divided them into nine different categories [41].

#### Secretion of exosomes

**Figure 1.**
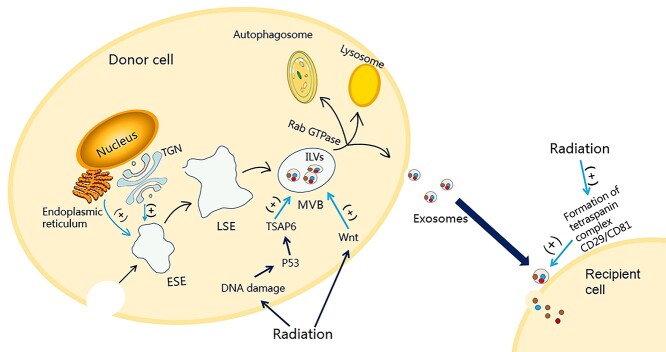
The effect of radiation on the biogenesis of exosomes. Exosomes originate from the invagination of the plasma membrane, forming ESEs, LSEs and finally forming MVBs containing multiple ILVs. The TGN and endoplasmic reticulum can promote the formation of ESEs. MVBs can fuse with the plasma membrane to free the contained ILVs as exosomes and fuse with lysosomes or autophagosomes to be degraded. Radiation can affect the production and secretion of exosomes, and the formation of tetraspanin complex CD29/CD81 increases after radiation, which leads to an increase in the uptake of exosomes by cells. *ESEs* early sorted endosomes, *LSEs* late-sorting endosomes, *MVBs* multivesicular bodies, *ILVs* intraluminal vesicles, *TGN* trans-Golgi network, *TSAP6* tumour suppressor-activated pathway 6

In MISEV2018, exosomes are proposed as a subtype of extracellular vesicles (EVs) [42]. Exosomes are endogenous extracellular lipid bilayer vesicles that can be secreted by almost all types of cells [28]. The process of exosome production includes the double invagination of the plasma membrane and the creation of intracellular multivesicular bodies (MVBs) carrying intraluminal vesicles (ILVs) [12] (Figure 1). The plasma membrane is invaginated for the first time to produce a cup-like structure, leading to the reformation of the early sorted endosomes (ESEs), which in some circumstances may be directly incorporated into the preexisting ESEs [12]. Furthermore the formation of ESEs increases with the help of the trans-Golgi network (TGN) as well as the endoplasmic reticulum [36,43]. ESEs can become late-sorting endosomes (LSEs) through inward invagination of the restrictive membrane of the endosome and finally generate MVBs, and MVBs contain multiple ILVs (future exosomes) [12]. Then through the fusion of MVBs to the plasma membrane and exocytosis, ILVs are finally secreted in the form of exosomes with a diameter of ~40–160 nm (average 100 nm) [12]. The density of exosomes in the sucrose gradient is 1.15–1.19 g/mL [44]. The MVBs can fuse with the plasma membrane to free the carried ILVs as exosomes and can be degraded by fusion with lysosomes or autophagosomes [36,45]. It is worth noting that the mechanism of MVB transport and fusion with the cell membrane is affected by some Rab guanosine triphosphatase (GTPase) proteins [46,47]. Besides, different cell types, cell states and culture environments may change the secretory mechanism of exosomes [48].

#### Components of exosomes

According to high-throughput research, exosomes contain a large number of bioactive substances, such as proteins, metabolites, mRNA [23], miRNA [23] and other non-coding RNA [49], mitochondrial DNA [50], and so on (Table 1). In addition, three exosome databases (ExoCarta, EVpedia and Vesiclepedia) provide relevant internal molecular information [51–53]. The main protein constituents of exosomes include membrane transport proteins and fusion proteins (Rab GTPases, annexins and flotillins, etc.), as well as protein families mostly associated with lipid microdomains, such as integrins and tetraspanins (such as CD63, CD9, CD81 and CD82) and heat shock proteins (such as hsp70 and 90) [54–56]. Bellingham *et al*. [57] conducted a small RNA deep-sequencing analysis of exosomes, which showed that exosomes carry various RNA species, including messenger RNA fragments, miRNA and other small non-coding RNA, retroviral RNA repeat regions, structural RNA, small interfering RNA, etc. In addition, it is found that the content of some miRNAs in exosomes is higher than that in cells, which indicates that some miRNAs can be specifically packaged in exosomes [23]. As far as lipid species are concerned, exosomes are rich in long acyl chains of saturated fats, which are often found in lipid raft components (e.g. cholesterol, ceramide, sphingomyelin as well as glycerol phospholipids) [58], so exosomes generally have more cholesterol, ceramide and sphingomyelin than the donor cells [59].

**Table 1:** Components of exosomes

**Content**	**Main components**	**References**
	Membrance transport proteins	
	Fusion proteins	
	Integrins	
	Transmembrane proteins,such as CD63, CD9, CD81, CD82	[54–56]
	Heat shock proteins, such as hsp 70 and 90	
	mRNA	
	miRNA	
	Other small non-coding RNA	
	Structural RNA	[57]
	siRNA	
	Retroviral RNA repeat regions	
	Cholesterol	
	Ceramide	
	Sphingomyelin	[58]
	Phospholipids	
Mitochondrial DNA		[50]

The miRNA and protein in exosomes can efficiently promote tissue injury repair [5]. Previous studies have demonstrated that metallothionein-2 transported by exosomes has anti-inflammatory effects on macrophages and mouse colitis models [53], and metallothionein-2 is a critical regulator of NF-κB signalling [60]. Moreover, miR-223-3p [61] and miR-126 [62,63] have been found to inhibit pro-inflammatory responses. Although there is an increasing number of reports about the composition of exosomes, the properties and functions of the molecules carried by exosomes under specific conditions need to be studied further.

#### Diagnostic potential of exosomes

Exosomes are present in almost all biological fluids. The biogenesis of exosomes involves complicated extracellular and intracellular molecular cargo that can be used for diagnostic testing [12] and provide a non-invasive or minimally invasive diagnosis [34]. miRNA, protein and lipid of exosomes can serve as diagnostic markers [64]. The specific miRNAs carried in exosomes can diagnose cancer or indicate the potential prognosis [65]. There is increasing evidence that exosomes derived from tumours carrying miRNA can be considered biomarkers for early cancer diagnosis [66]. In addition, the application of exosomes as a diagnostic tool can also be used in neurodegenerative diseases [67], cardiovascular diseases [68,69], liver diseases [70], kidney diseases [71], lung diseases [72], etc. Recent studies have shown that miR-125b in serum exosomes can be used as a non-invasive diagnostic marker for the severity of asthma [73].

Radiation can affect exosome composition, which can be used as a diagnostic biomarker to predict the effect of radiotherapy in patients [74]. Some studies have shown that exosomal miR-339-5p can improve esophageal squamous cell carcinoma radiosensitivity by down-regulating Cdc25A [75]. By analyzing the proteins of urine and serum exosomes in irradiated mice, it was found that urine-derived exosomes can reflect radiation injury to the liver and gastrointestinal and genitourinary tracts, and serum-derived exosomes can indicate radiation-induced vascular injury and acute inflammation [76]. Combining the contents of exosomes such as protein, lipid, RNA and miRNA, a multi-component combination method using combinatorial markers can be used to reflect the different aspects of disease-generating exosomes (such as metabolites, RNA and protein content), which can potentially improve the specificity and sensitivity of the diagnosis provided by exosomes [12].

### Effects of radiation on exosomes

#### The effect of radiation on exosome secretion

Research by Arscott *et al*. [77] showed that radiation enhanced the number of exosomes derived from glioblastoma cells and normal astrocytes. Furthermore, radiation can promote the release of exosomes derived from prostate cancer [78]. More and more evidence has shown that the release of exosomes induced by IR increases in a dose- and time-dependent manner, owing to the activation of additional pressure-induced exosome secretion pathways [78]. Researchers have explored several molecular mechanisms that influence exosome secretion (Figure 1). Previous studies have shown that induced secretion of exosomes in irradiated cells is regulated by tumour suppressor-activated pathway 6 (TSAP6) protein, and p53 regulates TSAP6 protein transcription, so the cellular state of this DNA damage response factor influences the composition and secretion rate of exosomes [79]. Lespagnol *et al*. [80] have proved that the production and secretion of exosomes are strictly regulated biological processes dependent on TSAP6. In human prostate cancer cell lines [78], human keratinocytes HaCaT cells [81] and breast epithelial cancer MCF7cells [82], it was confirmed that the exosomes secretion rate of p53 active cells increased after irradiation. Recent studies have demonstrated that low-level laser irradiation at high power intensity enhances the secretion of endothelial exosomes by activating transcription factors associated with Wnt signalling and autophagy stimulation [83]. In addition, it was found that the mitogen-activated protein kinase (MAPK) signalling pathway can regulate the secretion of exosomes [84]. Future research needs to explore further the secretion mechanism and influencing factors of exosomes to reveal the mechanism of influence of radiation on exosome secretion.

#### The effect of radiation on exosome composition

Regarding radiation-induced changes in the composition of exosomes, the current reports are mostly related to the proteome of exosomes. (Table 2). It was initially reported that B7-H3 (CD276) protein levels had risen in exosomes derived from irradiated prostate cancer cells [78]. By analyzing the exosomes secreted by FaDu cells derived from human squamous head and neck cell carcinoma, it was found that the proteins overexpressed in the exosomes of irradiated cells included those involved in transcription, translation, cell division and cell signal transmission [85]. In addition, the levels of connective tissue growth factor mRNA and insulin-like growth factor binding protein 2 protein carried by exosomes derived from irradiated glioblastoma were significantly increased [77]. Shen *et al*. [86] found that the expression level of 15 miRNAs in melanocyte-derived exosomes irradiated with ultraviolet B (UVB) was higher than that of melanocyte-derived exosomes not irradiated with UVB, confirming for the first time that UVB irradiation can enhance melanocytes’ secretion of exosomes and change their exosomal miRNA profile. Analysis of the effect of radiation on the composition of exosomes released from various cell types and radiation patterns shows that radiation can affect the secretion of the exosome, and in particular its molecular composition.

**Table 2:** Effects of radiation on the composition of exosomes

**Source of exosomes**	**Radiation source**	**Changes in the composition of exosomes**	**Reference**
Human prostate cancer cell	Ionizing radiation	Rise in level of B7-H3 (CD276) protein	[78]
Human squamous head		Rise in proteins include proteins involved in	
and neck cell carcinoma	Ionizing radiation	transcription, translation, cell division and cell	[85]
(FaDu)		signal transmission	
		Rise in level of insulin-like growth factor binding	
Human glioblastoma	Ionizing radiation	protein 2 proteins and connective tissue growth	[77]
		factor mRNA	
Human melanocyte	Ultraviolet B exposure	Rise in level of 15 miRNAs (miR4488, miR-320d,	[86]
		miR-7704 etc.)	

#### Radiation increases the cellular uptake of exosomes

Radiation exposure can affect the uptake of exosomes by cells (Figure 1). After being released into the extracellular environment, exosomes are absorbed by nearby and distant receptor cells to fulfill their regulatory functions. Exosome uptake includes at least three steps: binding to the cell surface, fusion with the plasma membrane and internalization of the receptor cell [87]. The interaction between exosomes with their receptors is through adhesion molecules and ligands situated on the surface of the bilayer membrane [88,89]. Then they are internalized, and their cargoes are released into the cell, where they perform their regulatory functions. Previous studies have shown that the uptake of exosomes by cells is enhanced in a radiation dose-dependent manner, whereas the uptake of exosomes by cells induced by radiation does not depend on the type of recipient cells [29]. Moreover the formation of the tetraspanin complex CD29/CD81 increases after radiation, resulting in increased uptake of exosomes by cells [29].

### Exosomes and radiation injury

#### Exosomes and radiation-induced skin injury

##### The influence of irradiation on the skin

The skin is the largest organ of the human body and performs a series of significant biological functions, such as regulating body temperature, sensory perception, excretion, absorption and immunologic functions. The cutaneous epithelium is a tissue with a high rate of dividing cells [90], so the skin is extremely susceptible to radiation injury. It has been pointed out that ultraviolet radiation has two effects: inducing cell ageing and transforming skin melanocytes into melanoma [91]. In addition, cutaneous radiation injury can also be classified as acute (early effect) and chronic (late effect). Marcu [92] revealed that early effects are more apparent in quickly proliferating tissues (e.g. skin and mucosa), while late effects occur in tissues with slow cell turnover. Types of acute cutaneous radiation injury include erythema, hyperpigmentation, dry desquamation, alopecia, moist desquamation and ulceration [93,94]. Chronic adverse effects tend to be underestimated and include delayed ulcers, fibrosis, ischemia, atrophy and cutaneous malignant changes [94–96]. Accumulating studies have shown that late effects are related to an imbalance of proinflammatory and profibrotic cytokines [96]. On the other hand, the risk of non-melanoma skin cancer increases after several years of radiation exposure [97]. In particular, cancers developed from keratinocytes (basal cell carcinoma, keratoacanthoma and squamous cell carcinoma) are radiation-related skin tumours [98,99].

##### The role of exosomes in radiation-induced skin injury

**Figure 2.**
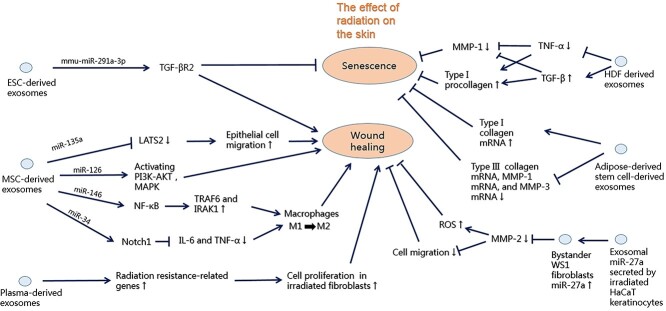
The role of exosomes in radiation-induced skin damage. Mmu-miR-291a-3p derived from ESCs can restrain cell senescence through the TGF-βR2 signalling pathway and accelerate the healing process of skin incisions in elderly mice. MSC- exosomal miR-135a increases epithelial cell migration by inhibiting the expression of the gene encoding LATS2 during skin wound healing. MSC-exosomal miR-126 can activate PI3K-Akt and MAPK pathways and promote the skin healing process. MSC-exosomal miR-146 up-regulates the expression of M2- related genes (such as TRAF6 and IRAK1) by targeting NF-κB signals, and miR-34 targets Notch1 to inhibit transcriptional pro-inflammatory cytokines encoding M1-related genes, such as IL-6 and TNF-α. Plasma-derived exosomes can promote wound healing by enhancing the expression of radiation resistance-related genes and regulating cell proliferation and ferroptosis of fibroblasts after radiation. In nude mice with ultraviolet B-induced skin photoaging, the exosomes derived from HDF have anti-skin senescence properties by down-regulating TNF-α and up-regulating TGF-β, causing the expression of type I procollagen to increase and the expression of MMP-1 to decrease observably. In a photoaging skin model of rats induced by ultraviolet B radiation, exosomes from adipose-derived stem cells increased the expression of type I collagen mRNA and decreased mRNA expression, including type III collagen, MMP-1 and MMP-3, thus significantly improving the photoinjury of skin. Irradiated HaCaT keratinocytes secrete exosomal miR-27a, which up-regulates the expression of miR-27a in bystander WS1 fibroblasts, resulting in decreased MMP-2 expression, which delays cell migration and increases ROS level, resulting in prolonged wound healing time. *ESC* embryonic stem cells, *TGF-βR2* transforming growth factor-beta receptor 2, *MSC* mesenchymal stem cell, *LATS2* large tumour suppresser homolog 2*, MAPK* mitogen-activated protein kinase, *TRAF6* tumour necrosis factor receptor-associated factor 6, *IRAK1* interleukin-1 receptor associated kinase 1, *IL6* interleukin 6, *TNF-α* tumour necrosis factor-alpha, *TGF-β* transforming growth factor-beta*, HDF* human dermal fibroblast, *MMP* matrix metalloproteinase, *ROS* reactive oxygen species

The role of exosomes in radiation-induced skin injury is summarized in Figure 2. Studies have shown that the proliferation and migration of skin fibroblasts affect the process of wound healing [100]. In the senescence of human dermal fibroblasts induced by IR, it was found that mmu-miR-291a-3p derived from embryonic stem cells (ESC) restrained cell senescence through the transforming growth factor-β receptor 2 (TGF-βR2) signalling pathway, and mmu-miR-291a-3p accelerated the healing process of a skin incision in aged mice [101]. In addition, mesenchymal stem cells (MSCs) play a crucial role in regenerative skin processes [102]. Increasing evidence has shown that the therapeutic activity of adult stem cells occurs partially through paracrine effects [103]. Exosomes are vital components that account for the paracrine action of MSCs [48,104]. Recent studies suggest some special roles of MSC-exosomal miRNAs in mediating epithelial recoveries, such as that of miR-135a in aiding epithelial cell migration by inhibiting expression of the gene encoding large tumour suppresser homolog 2 (LATS2) during skin wound healing [105] and that of miR-126 in activating the PI3K-Akt and MAPK pathways during cutaneous repair in a rat model of diabetes [106]. Furthermore, some studies have shown that NF-E2-Related Factor2 (Nrf2) knockdown suppressed the antioxidant capacities of MSC-exosomes *in vitro* and *in vivo*. Therefore, MSC-derived exosomes protect against oxidative stress-induced cutaneous injury by adaptive regulation of the Nrf2 defence system [107]. MSC-exosomes exert an immunomodulatory function, primarily by regulating the function of immune cells or changing their inflammatory cytokine secretion characteristics [108]. In the presence of interferon-γ (IFN-γ) and tumour necrosis factor-α (TNF-α), MSCs produce exosomes that induce macrophages to transform from M1-to M2-like phenotype, and exosomal miRNAs are involved in this process, such as miR-146 and miR-34 [109]. Mechanistically, miRNA-146 upregulates the expression of M2-related genes [tumour necrosis factor receptor-associated factor 6 (TRAF6) and interleukin-1 receptor associated kinase 1 (IRAK1)] via targeting Nuclear factor-kappa B (NF-κB) signals [110], while miR-34 targets Notch1 to inhibit the transcription of genes encoding M1-related pro-inflammatory cytokines, such as interleukin (IL)-6 and TNF-α [111]. In addition, research has shown that MSC-exosomes also suppresses epithelial–mesenchymal transition (EMT) by reducing epithelial depletion due to transformation, thereby maintaining the integrity of the epithelium and inhibiting tissue fibrosis [112,113]. Recent research has shown that plasma-derived exosomes can promote the healing of irradiated wounds by intensifying the expression of radiation resistance-related genes and regulating the cell proliferation and ferroptosis of fibroblasts after radiation [114].

In nude mice with UVB-induced skin photoaging, exosomes derived from 3D cultured human dermal fibroblast spheres showed anti-skin senescence properties by down-regulating TNF-α and up-regulating TGF-β, leading to an observable increase in the expression of type I procollagen and a decrease in matrix metalloproteinase (MMP)-1 [115]. Furthermore, Liang *et al*. [116] found that in a photoaging skin model of rats induced by UVB radiation, the injection of exosomes from adipose-derived stem cells significantly reduced the epidermal thickness and enhanced the dermis thickness of the photoaging skin, reduced the ratio of the stratum corneum in the epidermis, increased the expression of type I collagen mRNA and also decreased the expression of mRNA, including type III collagen, MMP-1 and MMP-3, thus significantly improving the photoinjury of skin.

The RIBE is defined as a biological response in nonirradiated cells receiving signals from other cells directly exposed to IR [117]. Exosomes can be used as a means of intercellular communication. Irradiated HaCaT keratinocytes secrete exosomal miR-27a, which up-regulates the expression of miR-27a in bystander WS1 fibroblasts, resulting in decreased MMP2 expression, which delayed cell migration and increased ROS level [118]. In addition, Tan *et al*. [118] also observed that the wound healing time was prolonged and the epidermis was thickened in mice due to subcutaneous injection of exosomes derived from irradiated HaCaT keratinocytes. Thus, it is suggested that RIBEs may play a potential role in wound healing. Therefore, how exosomal miRNAs affect bystander effects by targeting downstream genes needs further research [119]. Exosomes can promote skin cell proliferation and epithelial regeneration and participate in the immune regulation of damaged skin, thereby accelerating wound healing and delaying skin aging. However, the more extensive mechanisms of exosomes in radiation-induced skin injury need to be studied further.

#### Exosomes and radiation-induced lung injury

##### The influence of irradiation on the lung

The lung is one of the most sensitive organs to IR, and radiation-induced lung injury (RILI) is a familiar complication in thoracic tumour radiotherapy [4]. The influences of lung irradiation are typically divided into early radiation pneumonitis, occurring within days to a few weeks after radiotherapy, and late radiation-induced pulmonary fibrosis, occurring months to years after the radiotherapy, which includes tissue fibrosis, necrosis, atrophy and vascular damage [4,120]. Human alveolar epithelium comprises type I and types II lung cells, which account for 90 and 10% of alveolar cells [120]. It has been reported that radiation immediately induced EMT in type II alveolar epithelial cells through the extracellular regulated protein kinases (ERK)/glycogen synthase kinase-3β (GSK3β)/Snail signalling pathway [121]. Radiation can induce the production of ROS and nitrogen species (NGS), leading to DNA strand breaks and alveolar epithelial cell death [122]. In addition, the release of multiple cytokines is regarded as having a significant role in the pathogenesis of RILI [123,124], including proinflammatory cytokines such as IL-1α, IL-1β, IL-3, IL-6, IL-7, TNF-α and pro-fibrogenic cytokines such as TGF-β1 [125]. The persistence of the inflammatory state can develop into irreversible late radiation lung fibrosis. Advanced radiation pulmonary fibrosis is a chronic, progressive and ultimately fatal interstitial lung disease with a poor prognosis and poor response to existing drug treatments [126]. The pro-fibrogenic cytokine TGF-β is the primary driver of late radiation-induced pulmonary fibrosis. The increase in TGF-β levels after radiotherapy is accompanied by elevated expression of the type IV collagen gene [127]. TGF-β inhibits collagen catabolism by the stimulation of tissue inhibitors of metalloproteinases (TIMPs), which results in collagen accumulation and transformation of fibroblasts into myofibroblasts, further leading to increased expression of alpha-smooth muscle actin (α-SMA) and lung structural remodelling [128]. In addition, the enhanced activity of TIMPs and reduced MMP activity results in excessive extracellular matrix deposition [129] and excess collagen [122]. Interestingly, numerous studies suggest that a massive infiltration of alveolar macrophages was also observed in the lung after radiation [130,131]. Macrophages and fibroblasts are activated by ILs, TGF, TNF and platelet-derived growth factors, leading to the secretion of high levels of ILs and TNF-α through activated macrophages. TNF-α mediates radiation pneumonitis and fibrosis by inducing the expression of intercellular adhesion molecules and the production of prostaglandins, as well as other inflammatory mediators [132]. Moreover, Park *et al*. [133] showed that IR promotes EMT in lung epithelial cells by M2 macrophages secreting high levels of TGF-β. These changes result in lung fibrosis, which further causes the loss of respiratory capacity, tissue atrophy and necrosis [134].

##### The role of exosomes in radiation-induced lung injury

The toxic side-effects of radioprotectors, such as nausea, vomiting, diarrhoea and hypotension, limit their clinical use [10,11]. Biological growth factors and cytokines such as IL-7, IL-11, granulocyte-colony stimulating factor, macrophage-colony stimulating factor and keratinocyte growth factor have been used to mitigate radiation-induced injury. Nevertheless, the successful effects of these compounds are also limited [11]. Moreover, although lung transplantation is the most useful measure for treating radiation-induced pulmonary injury, it is limited due to the lack of available donated lungs and transplantation-related complications [135]. Therefore, we urgently need a more effective treatment strategy based on the pathological mechanism of RILI.

MSCs, as multipotent stem cells, can regulate inflammation reaction, facilitate survival and repair impaired resident cells and increase the regeneration of damaged tissues [136], indicating that MSCs are a promising drug candidate for the treatment of RILI. Bury *et al*. [137] found that MSC grafts significantly reduced the presence of pro-inflammatory macrophages and neutrophils, attenuated the innate inflammatory response and promoted bladder tissue regeneration. Indeed, growing evidence shows that the therapeutic effects of MSCs can be attributed to their capacity to secrete paracrine factors [138]. Exogenously used MSCs may exert their complex paracrine anti-inflammatory, anti-fibrosis and reproductive effects through the released EVs [139]. The release of various cytokines plays a major role in the pathogenesis of RILI, as mentioned above. Exosomes derived from MSCs can reduce the pro-inflammatory cytokines IL-1β, IL-6 and TNF-α and promote the production of high levels of anti-inflammatory IL-10 [140] (Table 3). In addition, researchers have found that mesenchymal stem cell-derived exosomes can enhance IL-10 and TGF-β1 in human peripheral blood mononuclear cells and facilitate the proliferation and immunosuppression of T lymphocytes. Xiong *et al*. [141] further revealed that T lymphocytes could promote EMT and accelerate the occurrence of radioactive pulmonary fibrosis, and the effect of β-catenin was a possible mechanism. Interestingly, the reports of Blazquez *et al.* [142] confirmed that exosomes derived from human adipose MSCs could suppress the differentiation and activation of T cells and decrease the production of IFN-γ by stimulating T cells *in vitro*. Also, the work of Moon *et al.* [143] demonstrated that exosomes derived from lung epithelial cells deliver caspase 3 (a pro-apoptotic factor) and activate macrophages through Rho-related coiled-coil kinase I. Therefore, clearing away lung epithelial cell-derived exosomes from the blood can reduce lung injury *in vivo*. Similarly, exosomes derived from lung macrophages release IL-36γ (a pro-inflammatory cytokine), leading to bacterial lung injury. Bone marrow mesenchymal stem cell (BM-MSC)-derived exosomes observably reduced the rate of apoptosis and suppressed the production of ROS after oxidative stress injury [113]. Exosomes derived from mouse mastocytes under oxidative stress can protect the recipient cells by improving their resistance to oxidative stress [144]. Moreover, MSC-exosomes also inhibit the EMT, as previously described. EMT is critical in inducing tissue fibrosis, leading to pathological rather than functional restoration of injured tissue.

**Table 3:** The role of exosomes in radiation-induced lung injury

**Exosome source**	**Biological functions**	**Exosomes containingmi RNAs**	**Immunoregulation effects**	**References**
MSC	1.Reduce levels of the pro-inflammatory cytokines (IL-1β,IL-6,TNF-α) and promote the production of the anti-inflammatory IL-10.	miR-181c	Reduce levels of IL-1β，TNF-α and induce high levels of IL-10 by targeting the TLR4/p65 signaling pathway.	[112, 137, 140, 150，^152^-^154^, ^156^]
	2. Enhance IL-10 and TGF-β1 in human peripheral blood mononuclear cells.	miR-let-7b	By targeting TLR4	
	3. Faciliate the proliferation and immunosuppression of T lymphocytes.	miR-21, miR-23a, miR-145	By targeting TGF-β2	
	4. Reduce the presence of pro-inflammatory macrophages and neutrophils.	miR-125b	By targeting Smad2	
	5. Protected type II alveolar epithelial cells against apoptosis by down-regulating serum amyloid A3 (SAA3).	miR-let-7c	By targeting TGF-βR1	
	6. Inhibit the epithelial-mesenchymal transition(EMT).	miR-30b-3p	Protect type II alveolar epithelial cells	
BM-MSC	Reduced the rate of apoptosis and supressed the production of ROS after oxidative stress injury.	miR-214	Alleviate cell oxidative stress damage	[113, 147]
Human adipose MSC	Suppress the differentiation and activation of T cells,and decrease the production of interferon-γ by stimulated T cells.	-	-	[142]
Lung epithelial cells	Deliver caspase 3 (a pro-apoptotic factor) and activate macrophages through Rho-related coiled-coil kinase I.	-	-	[143]
Lung macrophages	Further aggravate lung damage by release IL-36γ.	-	-	[143]
Mouse mastocytes	Protect the recipient cells under oxidative stress by improving the resistance of the recipient cells to oxidative stress.	-	-	[144]

*IL* interleukin, *TGF-β* transforming growth factor-beta, *SAA3* serum amyloid A3, *EMT* epithelial-mesenchymal transition, *TNF-α* tumour necrosis factor-alpha, *TLR4* toll-like receptor 4, *TGF-βR1* transforming growth factor-beta receptor 1, *ROS* reactive oxygen species

Accumulating studies have reported the changes of miRNA in lung cancer patients after radiotherapy [145,146], suggesting that miRNA may play a significant role in the pathological process of RILI. BM-MSC-derived exosomes can alleviate cell oxidative stress damage by releasing miR-214, but over-expression of miR-214 can further promote fibrosis [147]. Studies have shown that the use of MSC-derived exosomal miRNAs can treat various inflammatory diseases by regulating levels of the pro-inflammatory cytokines IL-1β, IL-6, TNF-α and others [148]. Exosomal miR-146a can be a key regulator of the innate immune response to prevent the expression of several pro-inflammatory factors, including TNF-α, IL-6 and IFN-γ, and direct the inflammatory response [149]. Similarly, MSC exosome-shuttled miR-181c can reduce levels of IL-1β and TNF-α and induce high levels of IL-10 by targeting the toll-like receptor 4 (TLR4)/p65 signalling pathway [150]. Moreover, enhancing the transcription of miR-146 in macrophages and monocytes can attenuate the activation of monocytes/macrophages and inhibit the response of pro-inflammatory macrophages, thereby suppressing NF-κB-mediated inflammation [151]. Indeed, growing evidence has suggested the modulation of expression levels of inflammatory cytokines in damaged tissue by MSC release of exosomal miRNAs, including miR-let-7b targeting TLR4 [152], miR-21, miR-23a and miR-145 targeting TGF-β2 154, miR-125b targeting Smad2 [153] and miR-let-7c targeting TGF-βR1 [154]. Recent research shows that the highly abundant miRNAs shuttled by human umbilical cord mesenchymal stem cells play a significant role in preventing inflammation and fibrosis [155], strongly suggesting the potential of MSC-released miRNAs for RILI therapy. Furthermore, MSC-exosomes promote the proliferation and survival of alveolar epithelial cells. For example, in an acute lung injury model, exosomes from miR-30b-3p overexpressing MSCs protected type II alveolar epithelial cells from apoptosis by down-regulating serum amyloid A3 [156]. In general, exosomes alleviate radiation-induced lung injury mainly by decreasing cellular oxidative stress damage, inflammation and fibrosis.

#### Exosomes and radiation-induced bone injury

##### The influence of irradiation on bone

The bone marrow is highly sensitive to radiation and is the major site of injury in radiotherapy [157]. Radiation-induced bone marrow injury leads to myelodysplasia, which is due to the death of progenitor cells, resulting in the loss of functional cells [158]. Sub-lethal doses of radiation can lead to bone marrow suppression and cause immunosuppression in patients due to abnormal numbers of functional blood cells. High doses of radiation can cause bone marrow failure. Radiation directly affects the differentiation potential of BM-MSCs and hematopoietic stem cells (HSCs) [159], leading to an overall decrease in the number of cells in the bone marrow and changes in bone marrow phenotypic composition [158]. This will not only cause immune system impairment but also weaken bone architectural quality. The bone marrow is an important part of the bone microenvironment because it produces MSCs and HSCs, which can be differentiated into osteoblasts (OBs) and osteoclasts (OCs), respectively [160]. EVs derived from mouse BM-MSCs can alleviate the radiation injury of bone marrow hematopoietic cells; EVs derived from endothelial cells can improve bone marrow cellularity, HSC and progenitor cell content in irradiated mice (reviewed in [161]). MSC-exosomes can repair radiation-induced hematopoietic system injury (reviewed in [5]). In bone, the tight regulation between bone formation by OBs and bone resorption by OCs is indispensable to maintain a functional skeletal system [162]. At a cellular level, OBs and adipocytes originate from the same progenitor cells, BM-MSCs, and can differentiate into various cell lineages [163]. Different from OBs, OCs are derived from HSCs in the bone marrow [159]. Numerous studies suggest that high IR doses primarily have deleterious effects on the bone, increasing bone resorption and decreasing bone formation [159]. Previous studies have shown that radiation-induced bone injury is mainly caused by defects in OBs that form bone. In addition, IR was reported to induce cell cycle arrest in OBs [164].

As BM-MSCs exhibit self-renewal capacity, high proliferative and various differentiation potentials are critical in bone recovery following irradiation, to maintain homeostasis with osteogenesis and adipogenesis under physiological conditions [165]. Results in earlier studies have shown that expression of runt-related transcription factor-2 (RUNX2) and osterix transcription factor are the major determinants for the osteogenic differentiation of BM-MSCs, and the peroxisome proliferator-activated receptor-γ (PPAR-γ) transcription factor and the CCAAT/enhancer-binding protein family are vital factors driving the adipogenic differentiation of BM-MSCs [166,167]. Wang *et al*. [168] revealed that the expression levels of RUNX2, alkaline phosphatase (ALP) and osteocalcin were decreased following irradiation, but no obvious changes in the gene and protein expression levels of PPAR-γ were observed following irradiation. Therefore, BM-MSCs preferentially differentiate into adipocytes rather than OBs after irradiation, which finally results in fat accumulation and bone loss [169].

##### The role of exosomes in radiation-induced bone injury

BM-MSCs play a significant role in maintaining bone homeostasis, and altered proliferation and differentiation of BM-MSCs is one major reason for irradiation-induced bone loss [165,170]. In recent years, researchers have sought to discover how to decrease the injury to BM-MSCs and restore their differentiation capacity in order to alleviate irradiation-induced bone loss. Research has shown that BM-MSC-derived microvesicles/microparticles and exosomes could reinduce the expression of chondrocyte markers (type II collagen, aggrecan) in osteoarthritis-like chondrocytes and suppress catabolic [MMP-13 and recombinant a disintegrin and metalloproteinase with thrombospondin 5 (ADAMTS5)] and inflammatory (inducible nitric oxide synthase) markers [171]. Exosomes and microvesicles/microparticles were also shown to protect chondrocytes from apoptosis and suppress macrophage activation [172].

Exosomes are special secretory vesicles involved in the paracrine effects of MSCs, and research has demonstrated that exosomes play a repairing role to the same extent as mesenchymal stem cell transplantation (MSCT) [28,173]. In healthy [174] and osteoporotic [175] animals, exosomes have also been shown to stimulate stem cell differentiation [176] and facilitate the repair of bone defects. Zhang *et al*. [174] demonstrated that exosomes could markedly promote human bone marrow-derived mesenchymal stem cell (HBM-MSC) osteogenic differentiation through activating the PI3K/Akt signalling pathway. Zuo *et al*. [113] revealed that MSCT and exosomes derived from BM-MSC transplantation could reverse bone loss in rats following irradiation, and exosomes can alleviate radiation-induced injury and accelerate DNA repair in BM-MSCs after irradiation. Exosomes alleviate radiation-induced oxidative stress in BM-MSCs by increasing the expression of antioxidant-related proteins, such as catalase, superoxide dismutase 1(SOD1) and SOD2. In addition, the study also found that exosomes can restore the balance of adipogenesis and osteogenesis in irradiated BM-MSCs through activating the Wnt/β-catenin signalling pathway [113] (Table 4). BM-MSCs start differentiation after irradiation, but the differentiation tendency towards adipocytes exceeds that of OBs, and BM-MSC-derived exosome transplantation could restore the differentiation potential of irradiated BM-MSCs [113]. Because of its higher safety, low immunogenicity and easier storage, delivery and management, exosome therapy is a better option than MSC transplantation [13]. Moreover, the research of Xu *et al*. [177] revealed the presence of miRNAs in exosomes during BM-MSC osteogenic differentiation. They found that miR-218, miR-let-7a, miR-299-5p, miR-148a, miR-199b, miR-219 and miR-302b were up-regulated in exosomes derived from BM-MSCs. Nevertheless, miR-221, miR-155, miR-885-5p, miR-181a and miR-320c were markedly down-regulated in exosome specimens [177]. Mechanistically, miRNA and miR-let-7 can suppress the adipogenesis of HBMSCs by regulating the expression of the high mobility group A2 (HMGA2) gene, thereby promoting bone formation [178]. It has been reported that miR-218 can accelerate the osteogenic differentiation and mineralization of human adipose tissue stem cells by activating the Wnt/β-catenin signalling pathway [179]. MiR-885-5p expression down-regulates osteoblast activity by targeting RUNX2 [177]. miR-199b is also involved in the regulation of OB differentiation by RUNX2 [178]. miR-181a inhibits TGF-β signalling molecules through suppressing TGF-βR1/ALK5 and promotes OB differentiation and mineralization [180]. Furthermore, it has been found that exosomal miR-130b is overexpressed in both osteogenic and chondrogenic MSCs, indicating that miR-130b plays a vital role in the osteogenic and chondrogenic differentiation of MSCs [181].

**Table 4:** The role of exosomes in radiation-induced bone injury

**Exosome source**	**Effects on irradiation of bone tissue**	**Exosomes containing** **miRNAs**	**Biological functions**	**References**
BM-MSCs	1. Alleviate radiation-induced oxidative stress in BM-MSCs by increase the expression of antioxidant-related proteins (catalase,SOD1,SOD2).	miR-let-7	Supress the adipogenesis of hBM-MSC by regulating the expression of HMGA2 gene, thereby promoting bone formation.	[113, 177-180]
	2. Accelerate DNA repair in BM-MSCs after irradiation.	miR-218	Accelerates osteoblast differentiation and mineralization through Wnt signaling.	
	3. Restore the differentiation potential of irradiated BM-MSCs.	miR-199b	Involved in the regulation of osteoblast differentiation by Runx2.	
	4. Restore the balance of adipogenesis and osteogenesis of irradiated BM-MSCs by activating the Wnt/β-catenin pathway.	miR-885-5p	Inhibits osteogenic differentiation by targeting RUNX2.	
		miR-181a	Inhibit TGF-ß signaling molecules through suppressing TßR-I/ Alk5 and promotes osteoblast differentiation and mineralization.	
BM Stromal cells	Promte the bone remodeling process by increasing osteoblast differentiation and mineralization.	-	-	[182]
Mature osteoblasts	Promoted bone growth by up-regulating RUNX2 and alkaline phosphatase, as well as strengthened matrix mineralization.	miR-677-3p	Promotes MSC osteogenic differentiation via targeting axis inhibition protein 1.	[184-186]
		miR-378	Improves the glucose-mediated osteogenic differentiation through activating PI3K/Akt signaling pathway.	
		miR-335-5p	Enhances osteoblast differentiation and mineralization by reduce the expression of DKK-1.	

*BM-MSCs* bone marrow mesenchymal stem cells, *SOD* superoxide dismutase, *HMGA2* high-mobility group A2, *RUNX2* runt-related transcription factor-2, *TGF-βR1/ALK5* transforming growth factor-beta receptor type I, *MSCs* mesenchymal stem cells, *DKK-1* dickkopf-1

The recent work, Behera and Tyagi [182] reported that exosomes derived from bone marrow stromal cells could promote the bone remodelling process by increasing OBs. Bone morphogenetic protein 9, growth factors and TGF-β1 existing in exosomes derived from bone marrow cells can facilitate osteogenic differentiation [183]. In addition, exosomes from mature OBs also accelerated bone growth by enhancing RUNX2 and ALP and strengthened matrix mineralization [184]. Previous research also confirmed that miR-335-5p enhances OB differentiation and mineralization by reducing the expression of dickkopf-1 (DKK-1) [185]. Recent research shows that exosomal miRNAs are produced through OB mineralization and accelerate osteogenic differentiation [182]. MiR-677-3p enhances the expression of axis inhibition protein 1 and improves MSC osteogenic differentiation [184]. MiR-378 improves glucose-mediated osteogenic differentiation by activating the PI3K/Akt signalling pathway [186]. In radiation-induced bone injury, exosomes can restore the differentiation potential of BM-MSCs, alleviate oxidative stress and accelerate DNA repair, thus promoting osteogenic differentiation and repair of bone defects.

### Prospects and challenges of exosomes in radiation injury

At present, medical radiation countermeasures can be divided into three categories: radioprotective agents delivered before radiation exposure, radiation mitigators given shortly after radiation exposure but before the appearance of radiation symptoms and therapeutics applied after the onset of symptoms [187]. The Food and Drug Administration (FDA) approved amifostine as a radioprotector and chemoprotector in 1995 [188], which should be used before or at the time of radiation [189]. Amifostine is a free-radical scavenger, which can protect cells from IR damage when used before radiation exposure. Preclinical studies have shown that amifostine selectively protects normal cells, mainly by scavenging free radicals, accelerating the recovery of damaged DNA by donating hydrogen and inducing hypoxia in cells [188]. Amifostine has a good radioprotective effect. However, its application is severely limited because of its short half-life, delivery by injection, the lack of an oral formulation, poor compliance and severe adverse effects, including nausea, vomiting, hypotension and allergic reactions [190,191]. As the risk of radiation exposure increases, the development of new radioprotectors is essential. The therapeutic application of exosomes has great potential. Furthermore, the delivery of biologically active substances from exosomes to receptor cells effectively changes their biological responses. In radiation-induced injury, exosomes have been shown to play a non-negligible role in treating and preventing tissue injury, providing new insights for the treatment of radiation injury. However, in other radiosensitive organs such as the intestine and salivary gland, the role of exosomes needs further research. The high self-renewal rate of intestinal stem cells leads to the intestinal epithelium being highly sensitive to radiation [192]. Radiation-induced gastrointestinal syndrome is the result of a combination of direct cytocidal effects on the intestinal crypt and endothelial cells and subsequent loss of the mucosal barrier, leading to microbial infection, diarrhea, electrolyte imbalance, septic shock and systemic inflammatory response syndrome [193,194]. Recent studies have shown that macrophage-derived EV-packaged WNTs are critical for intestinal anti-radiation regeneration, which can reduce the radiation injury of intestinal epithelial cells and induce intestinal epithelial repair [192,195]. In addition, EVs derived from MSCs can reduce intestinal toxicity, promote epithelial repair and regeneration and maintain intestinal epithelium structural integrity in a mouse model of acute radiation syndrome [196]. Radiotherapy is the main treatment for head and neck cancer, but >75% of patients experience salivary gland niche destruction, salivary gland dysfunction and xerostomia after radiotherapy [197]. Because the salivary glands proliferate slowly and are composed of highly differentiated cells, they are sensitive to radiation [198]. Lombaert *et al*. [199] showed that salivary gland stem cell transplantation could rescue radiation-impaired salivary gland function. Transplantation of MSCs from bone marrow [200] and adipose tissue [201] can restore the morphology and function of the salivary glands in irradiated glands. In recent years, great progress has been made in research on a regeneration strategy for xerostomia. However, there are few reports on the role of exosomes in radiation-induced salivary gland injury, which needs to be further explored by researchers.

MSC-derived exosomes showed high safety in the treatment of refractory graft vs. host disease, repeated injections were well tolerated and no side effects were found [202]. Compared with cell therapy, exosomes have attractive advantages. Exosomes are considered the best candidates for cell-free therapy due to their ease of operation (resistant to freezing and thawing), high biological permeability and non-obvious immune rejection, and more importantly, their ability to cross the blood–brain barrier [28,203,204]. In addition, unlike fragile living cells, exosomes have a double-layer lipid structure that can maintain biological activity even after repeated manipulations [203]. Microvascular plugging or loss of transplanted cell viability hinders cell therapy, which is not a problem for exosome therapy [205]. Exosomes can be designed to deliver different therapeutic effective loads to the desired targets, such as short interfering RNA and chemotherapeutic drugs and immunomodulators [12]. Exosomes have good biocompatibility and can avoid being taken up by the reticuloendothelial system, which protects the encapsulated drug until it is delivered to the targeted site [19,206,207]. In addition, their bioavailability and ability to cross the blood–brain barrier makes exosomes promising carriers [208]. However, exosomes as the delivery vehicle for therapy need to be targeted. The components of exosomes derived from diverse cells are different, and their potential biological functions are also different. At present, most research on exosomes is still focused on protein classification, but their main function may be related to RNA delivery, so determining the mechanism of RNA sorting is beneficial to various applications of exosomes [28]. Moreover, research on the function of exosomes needs to be verified by more *in vivo* experiments.

In addition, the instability of the exosome contents is also an urgent problem. Some research has shown that the irradiation dose, as well as the pH value of the culture medium, can affect the number of miRNAs loaded in the exosome [209,210], and there is still a lack of unified standards for purification and quantification of exosomes from conditioned medium. The need to accurately describe exosomes will continue to rise as our understanding of the heterogeneity of exosomal cargo and function increases. The identification and isolation of single exosomes will substantially increase our understanding of the biological function of exosomes [12]. Therefore, we need to formulate more standardized purification techniques and analytical methods to study exosomes, and provide a theoretical basis for the correlation between exosomes and radiation injury and the application of exosomes in treatment and diagnosis.

## Conclusions

Exosomes are vital for intercellular communication and have a lot of promise for repairing radiation injury. As indicated above, in radiation-induced injury, exosomes can stimulate cell proliferation and regeneration, relieving cellular oxidative stress damage and regulating inflammatory responses and enhancing differentiation. Although there are some challenges in the clinical application of exosomes, as they receive increasing attention, researchers will gain a deeper understanding of exosomes, which will provide new ideas for their use in the treatment and prevention of radiation injury.

## Conflicts of interest

None declared.

## Authors’ contributions

SD and YW contributed equally to the review and should be viewed as co-first authors; they drafted and revised the manuscript. PL, LM and YL contributed to helpful discussion and reviewed the manuscript. JA and CS conceived and designed the manuscript, supervised the project and constantly gave invaluable feedback. All authors read and approved the final manuscript.
